# Multimodal non-invasive approaches for early Alzheimer’s disease detection: a review of neuroelectrophysiological and neuroimaging techniques

**DOI:** 10.3389/fpsyt.2026.1824430

**Published:** 2026-06-04

**Authors:** Linlin Lou, Yuji Zhou, Haohao Zhu, Kai Zheng, Yingying Ji

**Affiliations:** The Affiliated Mental Health Center of Jiangnan University, Wuxi Central Rehabilitation Hospital, Wuxi, Jiangsu, China

**Keywords:** Alzheimer’s disease, electroencephalogram, event-related potential, magnetic resonance imaging, transcranial magnetic stimulation-electroencephalogram

## Abstract

Alzheimer’s disease (AD) is a progressive neurodegenerative disorder characterized primarily by a gradual decline in cognitive function and specific pathological changes in the brain. In recent years, although various neuroelectrophysiological and neuroimaging techniques have greatly advanced the mechanistic study of abnormal brain function in AD, an integrative discussion of these technologies remains fragmented. This paper primarily summarizes and interactively analyzes the research progress of several non-invasive neuroimaging and neuroelectrophysiological techniques—event-related potential (ERP), electroencephalogram (EEG), transcranial magnetic stimulation-electroencephalogram (TMS-EEG), functional near-Infrared spectroscopy (fNIRS), magnetoencephalography (MEG), structural magnetic resonance imaging (structural MRI) and functional magnetic resonance imaging (fMRI)—to depict a panoramic view of AD pathology from a microscopic to a macroscopic scale from a multimodal perspective. It further compares the advantages and limitations of various technologies for detecting early AD biomarkers, emphasizing the synergistic value of multimodal integration in capturing changes in dynamic functional and structural brain networks. Additionally, we explore the potential of these technologies in clinical translation, particularly when combined with machine learning and deep learning approaches, to enhance the accuracy of early diagnosis and the depth of mechanism analysis. Through the above discussion, this review aims to provide new insights for the early identification of AD and advance our understanding of the neural mechanisms underlying AD.

## 1 Introduction

Alzheimer’s disease (AD) is the most common type of dementia globally, accounting for approximately 60-80% of all cases (1). Epidemiological evidence from global populations indicates that the number of individuals with AD and other dementias is continuously increasing, driven by population aging, posing a long-term and immense challenge to healthcare and long-term care systems. According to a study by Gustavsson et al., there are about 32 million people worldwide in the dementia stage of AD, 69 million in the biomarker-positive mild cognitive impairment (MCI) stage, and another 315 million in the preclinical stage. Combined, these groups represent about 22% of the population aged 50 and over, indicating a vast number of potential patients even before symptoms appear. The prevalence is generally higher in women than in men, with age being the primary risk factor (2). The incidence of AD increases with age, with reports ranging from approximately 5 to 16 cases per 1,000 person-years in different countries (4.9 in China for ages ≥55, 5.5 in Spain for ages ≥65, and 16.3 in Greece for ages ≥65). The five-year mortality rate for AD patients is about 35%, with registry data showing a mortality rate of 115 per 1,000 person-years, double that of their healthy peers. Furthermore, although the majority of patients reside in low- and middle-income countries, the per capita and total healthcare burden is more prominent in high-income nations (3).

AD has an insidious onset and a slow progression, making early detection and effective intervention critically important. Its classic pathological hallmarks include the extracellular deposition of Aβ plaques and the intracellular formation of neurofibrillary tangles (NFTs), accompanied by synaptic loss and progressive neurodegeneration (4). Research has found that these pathological changes begin years, or even decades, before the onset of clinical symptoms. This long developmental stage provides a critical window for early intervention but also presents significant challenges for diagnostic technologies. This implies that the earlier high-risk individuals are identified, the greater the potential for intervention before irreversible neural damage occurs (5). Numerous longitudinal studies have shown that approximately 10–15% of individuals with MCI progress to AD-type dementia each year (6). However, not all MCI patients will convert to AD, and the risk of conversion varies, with factors such as comorbid depression potentially accelerating the process (7). In their seminal review, Delbeuck et al. systematically defined AD as a progressive “disconnection syndrome.” This perspective has since become a core theoretical framework in modern AD research, widely cited and confirmed by numerous subsequent studies. This theory posits that the core pathophysiological mechanism of AD lies not only in the loss of neurons in specific brain regions but, more critically, in the interruption of communication and functional disintegration of the large-scale neural networks connecting these regions. This perspective emphasizes the need to understand and examine how the brain’s information processing system gradually fails from a multidimensional level (8).

However, the current “gold standard” biomarkers for AD diagnosis, such as cerebrospinal fluid analysis and PET imaging, while effective in reflecting Aβ and tau protein pathology, have inherent limitations including invasiveness, high cost, and low accessibility, making them difficult to meet the demands of large-scale early screening and longitudinal monitoring. Consequently, there is an urgent need for low-cost, repeatable, and non-invasive indicators of brain function for early screening (7, 9). Electroencephalography (EEG), a neurophysiological tool with millisecond-level temporal resolution, has shown unique advantages in studying AD and its prodromal stages. EEG can record synchronous neural firing at a 1ms scale in a subject’s natural state, making it suitable for capturing phenomena in MCI such as rhythm slowing, decreased phase synchronization, and reduced complexity (10). Furthermore, high-density EEG (HD-EEG) with 128–256 channels has significantly improved source localization accuracy, reducing localization errors in pathological regions like the temporal and parietal lobes to within 10mm, providing a solid structural basis for studying cortico-cortical connectivity patterns (11). The cost of a standard 30-minute EEG examination is typically less than one-tenth of a PET scan, making it a suitable method for large-sample longitudinal follow-up studies (12). Transcranial magnetic stimulation combined with EEG (TMS-EEG) is a non-invasive technique that synchronizes TMS with EEG. Researchers first stimulate the cortex and then record the brain’s potential waveforms with millisecond precision, allowing them to measure post-stimulus amplitudes and latencies and track signal propagation between different cortical regions (13). In new drug evaluations or neuromodulation trials, a single TMS-EEG session can provide real-time validation of whether an intervention has hit its target, substantially increasing the efficiency and sensitivity of early clinical development (14). Functional magnetic resonance imaging (fMRI) offers a significant advantage over EEG in spatial resolution, enabling the fine-grained depiction of whole-brain structural features and functional networks at the millimeter level. This spatial precision makes it invaluable for localizing and analyzing brain structure and function. A longitudinal resting-state functional magnetic resonance imaging (rs-fMRI) study by Malotaux et al. demonstrated that progressive MCI patients showed a progressive increase in functional connectivity within the default mode network (DMN) as the disease advanced, and the rate of this change correlated with cognitive decline, suggesting it could be an indicative network feature (15). In task-based functional magnetic resonance imaging (task-fMRI), Li et al. divided amnestic MCI (aMCI) patients into two subgroups based on Traditional Chinese Medicine (TCM) syndromes, namely “turbid phlegm clouding the orifices” (a literal translation of the TCM concept (tán zhuó méng qiào) and “spleen-kidney deficiency” (pí shèn kuī xū) and found distinct activation patterns during an episodic memory recognition task, providing objective neuroimaging evidence for the heterogeneity within aMCI (16). Additionally, the millimeter-level spatial resolution of fMRI allows for the observation and quantification of functional changes in deep brain structures like the striatum and thalamus. Hojjati et al. found that a multimodal machine learning model integrating graph theory metrics from rs-fMRI networks with structural MRI could predict the conversion from MCI to AD with an accuracy exceeding 0.90 (17, 18). Therefore, an integrative multimodal framework is crucial for comprehensively depicting the pathological panorama of AD.

**Figure 1:**
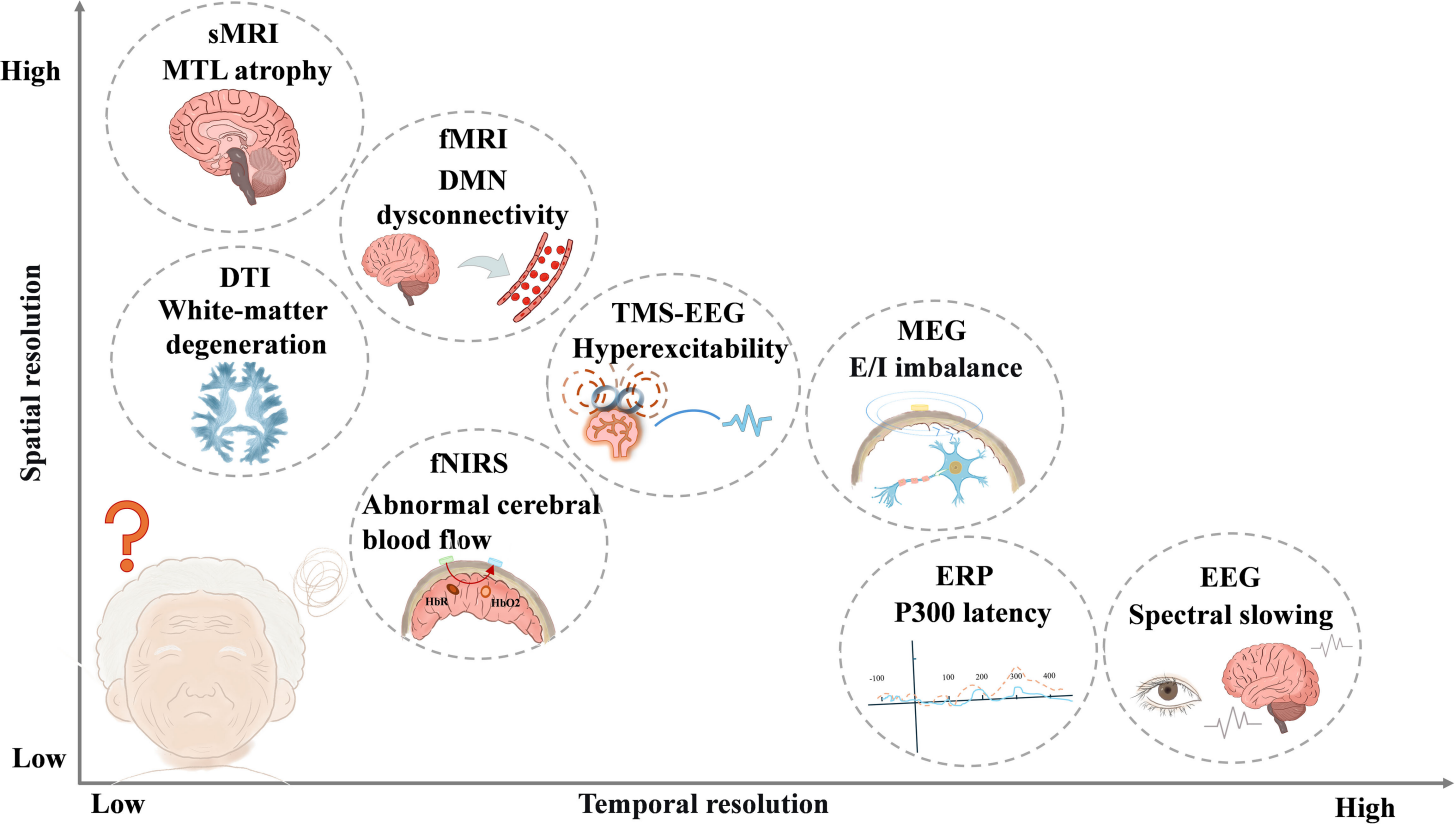
Multimodal data fusion on the AD continuum.

In summary, although research on imaging and electrophysiology in AD is rapidly advancing, information across different techniques remains fragmented. Most reviews tend to focus on a single modality or a single disease stage, failing to provide a systematic reference for clinical early screening and multimodal fusion research. Therefore, this review aims to achieve the goal of systematically delineating the typical changes during the progression of AD by integrating key findings from multimodal techniques such as ERP, EEG, TMS-EEG, MEG, structural MRI, fNIRS and fMRI. We will argue that these low-cost, repeatable, and complementary brain function indicators, when combined with advanced algorithms like machine learning, can not only provide practical solutions for early screening in resource-limited settings but also fundamentally deepen our understanding of the pathophysiological mechanisms of AD, laying a solid methodological foundation for future precision interventions and efficacy evaluations. **Figure 1** and **Table 1** show several complementary modalities converge on a fusion/ML hub to provide a synergistic diagnostic advantage: earlier diagnostic window, higher accuracy, and a more complete pathophysiological picture.

**Table 1:** Advantages and limitations of the eight modalities in the AD continuum.

Modality	Core advantages	Principal limitations
EEG	Excellent temporal resolution (ms-level); low-cost and scalable for longitudinal monitoring.	Poor spatial resolution and limited disease specificity.
ERP	High temporal resolution for tracking specific cognitive processes.	Weak spatial localization; requires extensive subject cooperation.
TMS-EEG	Unique ability to causally probe cortical excitability, connectivity, and plasticity.	High technical complexity and susceptibility to stimulation artifacts.
fMRI	High spatial resolution for mapping whole-brain functional networks.	Indirect measure of neural activity with low temporal resolution and high cost.
structural MRI-T1/T2	High-resolution, standardized anatomical marker of neurodegeneration.	Insensitive to early functional changes preceding macroscopic atrophy.
DTI	Provides *in-vivo* assessment of white matter microstructural integrity.	Indirect measure susceptible to fiber crossing and model-dependent metrics.
MEG	Direct measurement of neural activity with ms-level temporal resolution; higher spatial precision for cortical source localization than EEG due to minimal skull attenuation.	High cost, limited scalability, and strict requirement for a magnetically shielded room.
fNIRS	Non-invasive, highly portable, robust to motion artifacts, and higher temporal resolution than fMRI for capturing cortical hemodynamics.	Limited penetration depth (restricted to superficial cortical areas) and lower spatial resolution compared to fMRI.

We performed a systematic literature search up to October 2025 across PubMed, Web of Science, and Google Scholar. Our search queries combined topic keywords like “Alzheimer’s disease” and “mild cognitive impairment” with technical terms such as “ERP,” “fMRI,” “MEG,” “fNIRS,” “structural MRI,” “TMS-EEG,” and “DTI.” Our selection criteria prioritized longitudinal studies with prospective designs or large cohorts, systematic reviews, and meta-analyses from high-impact journals published in the last ten years. We also included seminal, highly-cited early literature foundational to the field. To ensure transparency and reproducibility, single-case reports and studies using non-standardized or non-reproducible paradigms were excluded. Throughout the review, each technical section critically examines the consistency and sources of heterogeneity in the evidence—including variations in paradigms, sample characteristics, and analysis pipelines—and addresses their limitations. This approach aims to deliver a review that is not only current but also structurally clear, evidence-driven, and critical.

## 2 Event-related potentials

### 2.1 Positioning and value of ERPs in AD research

Event-related potentials (ERPs) are specific electrophysiological responses generated by the central nervous system that are time-locked to specific sensory, cognitive, or motor events. They are an objective electrophysiological technique capable of dynamically tracking the process of information processing in the brain. This section, based on the classic Braak staging (19) and recent studies by Berron et al. and Therriault et al., posits that the pathological changes of AD begin in the hippocampus and entorhinal cortex of the medial temporal lobe (MTL) and progressively spread to the association cortex. Based on this theory, we propose that abnormalities in different ERP components during the course of AD should exhibit stage-specific characteristics: early pathological changes primarily affect components related to episodic memory retrieval, such as the P600/LPC. As the pathology gradually extends to the attentional and semantic processing networks of the neocortex, abnormalities in P300 and N400 also emerge and worsen (20, 21). To ensure the accuracy of our arguments, we have provided representative evidence for each ERP component discussed, excluding case reports or studies using non-standardized or difficult-to-replicate task paradigms. Concurrently, within each subsection, we will explicitly discuss the sources of heterogeneity and limitations present in current research.

### 2.2 Abnormal components at different processing stages

#### 2.2.1 Early impairment in pre-attentive and early sensory processing: MMN

Mismatch Negativity (MMN), due to its characteristic of not requiring active participation from the subject, can objectively reflect the brain’s predictive and error-correction processes for sensory information, making it considered one of the ideal tools for detecting early subclinical functional impairment (22, 23). The studies by Ruzzoli et al. and Özbayrak-Karapınar et al. found that reduced MMN amplitude and altered latency could be observed at the MCI stage, suggesting that the brain’s more fundamental sensory world-modeling capabilities may be compromised before a significant decline in the ability to perform complex cognitive tasks that require active engagement (22, 24). Research by Tsolaki et al. further demonstrated that as MCI progresses to AD, MMN and P300 latencies are generally prolonged and their amplitudes are reduced. Moreover, changes in activity related to pathological foci can be accurately localized, providing direct electrophysiological support for the early detection of brain function impairment (25). However, it is important to emphasize that the measurement of MMN is highly sensitive to various factors, such as stimulus modality, parameters, intervals, and the individual’s sensory system status (e.g., hearing level), leading to significant heterogeneity across studies. Therefore, current evidence supports MMN more as a potentially sensitive marker for detecting neural dysfunction in the very early stages of AD, even at the MCI stage, rather than as a standardized and stable prognostic biomarker.

#### 2.2.2 Decline in attentional allocation and working memory updating efficiency: P300

Among the numerous ERP components, the P300, particularly the P3b, is the most extensively studied cognitive ERP. This component is typically defined as a positive-going waveform occurring approximately 250–500 ms after the presentation of a target stimulus. Its amplitude primarily reflects the attentional resources allocated to the target, while its latency is associated with the duration of stimulus evaluation and decision-making, serving as a neural index of the efficiency of working memory updating. To fully understand its temporal dynamics, it is critical to distinguish between its two primary subcomponents: the frontocentral P3a and the centroparietal P3b. The P3a occurs slightly earlier and reflects a bottom-up, involuntary attentional orienting response to novel or distracting stimuli. In contrast, the P3b is associated with top-down, voluntary attentional allocation and the efficiency of working memory updating for task-relevant targets (26).

Research by Cecchi et al. (27) found that AD patients typically exhibit a P300 potential with prolonged latency and reduced amplitude. While both subcomponents are affected in the AD continuum, the robust attenuation of the P3b highlights the core deficit in working memory updating, whereas altered P3a dynamics may serve as an early indicator of impaired novelty processing and frontal network dysfunction. This change is already present at the MCI stage and worsens as the disease progresses, indicating a progressive decline in the efficiency of working memory updating and attentional resource reallocation. Multiple systematic reviews and meta-analyses have confirmed this conclusion in comparisons between MCI and healthy older adults, as well as between AD and healthy older adults (28–30), making it one of the most reliable findings in AD electrophysiological research. Consequently, the prolonged latency and reduced amplitude of the P300 are widely considered an important electrophysiological marker of early neural dysfunction in AD. A study by Jiang et al. further suggests that a P300 morphology with longer latency and lower amplitude at baseline is significantly associated with an increased risk of progression from MCI to AD, indicating its potential as a clinical warning and subtyping indicator (29). Although a study by Parra et al. using a classic visual oddball paradigm found that the P3b has moderate classification efficacy in discriminating MCI and predicting conversion from MCI to AD (31), the heterogeneity of its results cannot be ignored. This is mainly related to the choice of task paradigms and electrode sites, suggesting that future clinical applications will require standardized operational procedures. In summary, P300 latency can be regarded as a temporal metric of declining efficiency in cross-regional neural communication, highly consistent with the network-level disconnection hypothesis.

#### 2.2.3 Collapse of semantic and memory networks: N400/P600 (LPC)

The N400, a negative-going waveform peaking at approximately 400 ms, serves as a crucial neural index of lexical-semantic information integration. In semantic violation and semantic priming paradigms, AD patients typically exhibit a reduction in N400 amplitude or an abnormal topographical distribution as the disease progresses, reflecting the functional disruption of neocortical semantic networks (32). Meanwhile, the P600 (also known as the Late Positive Component, LPC), which is associated with episodic memory and encoding, shows a diminished or absent repetition effect even in the early stages of amnestic MCI (aMCI). A longitudinal study conducted by Olichney et al. (33) demonstrated the prognostic value of these components. The study found that if individuals with aMCI exhibited an abnormal or attenuated N400/P600 repetition effect at baseline, their probability of converting to AD within three years was as high as 87–88%. In contrast, the conversion risk for those with a preserved ERP repetition effect was only 11–27%. This risk disparity highlights how electrophysiological alterations map onto the anatomical progression of AD. Specifically, early P600 abnormalities primarily reflect localized synaptic dysfunction within the memory centers of the medial temporal lobe. The subsequent emergence of N400 deficits signifies that the pathology has advanced into the widespread semantic networks of the neocortex. This transition indicates that the entire cognitive system may have reached a tipping point of decompensation (34). As summarized in **Table 2**, ERP component alterations along the AD continuum reflect a hierarchical spread of pathology from the medial temporal lobe to neocortical networks, with early involvement of P600/LPC, followed by P300 and N400 abnormalities.

**Table 2:** Summary of representative ERP findings across the AD continuum.

ERP component	Cognitive domain/paradigm	Population	Main electrophysiological findings	Functional interpretation	Representative studies
MMN (Mismatch Negativity)	Passive auditory/visual oddball paradigm; pre-attentive sensory processing	CN, MCI, AD	↓ Amplitude; ↑ Latency (from MCI stage); source activity localized to fronto-temporo-parietal regions.	Reflects early impairment in automatic sensory prediction and error updating; potential preclinical marker of network-level disconnection.	Ruzzoli et al., 2016 (22); Tsolaki et al., 2017 (25)
P300 (P3b)	Auditory/visual oddball paradigm; attention and working memory updating	CN, MCI, AD	↓ Amplitude; ↑ Latency; effect size moderate–large; alterations appear in MCI and worsen in AD.	Indicates slowed information evaluation and distributed network communication; sensitive electrophysiological marker for cognitive decline and MCI→AD progression.	Cecchi et al., 2015 (27); Tarawneh et al., 2021 (28); Jiang et al., 2015 (29); Demirayak et al., 2023 (30); Parra et al., 2012 (31)
N400	Semantic violation/priming paradigms	CN, MCI, AD	↓ Amplitude; abnormal topography.	Semantic network disintegration; impaired lexical-semantic integration.	Olichney et al., 2008 (33); Joyal et al., 2020 (32)
P600/LPC	Word repetition/episodic retrieval paradigms	CN, aMCI, AD	Reduced or absent repetition effects even at early aMCI stage.	Early breakdown of hippocampal-dependent memory re-encoding; strong predictor of dementia conversion (87–88% vs 11–27%).	Olichney et al., 2008 (33)

### 2.3 Integration with machine learning

At the algorithmic level, machine learning and deep learning provide powerful analytical tools for maximizing the diagnostic information extracted from ERP and multimodal data (35). Unlike traditional analysis, which relies on predefined time windows and specific component peaks, deep learning models such as Convolutional Neural Networks (CNNs) can automatically learn complex spatio-temporal features related to the disease from raw, high-dimensional ERP or EEG data (36). For instance, a study by Liu et al. (37) achieved high-precision classification of AD by fusing EEG and structural MRI data with a deep learning model, reporting an accuracy of up to 94.7%. Duenias et al. (38) proposed a novel hypernetwork framework called HyperFusion, which dynamically adjusts the imaging analysis process by integrating tabular clinical data with MRI, thereby achieving predictive performance in tasks like Alzheimer’s classification that surpasses single-data-source approaches and other advanced fusion methods. These studies collectively confirm that multimodal data fusion is significantly superior to any single modality, providing a more comprehensive disease profile.

In predicting conversion and evaluating treatment efficacy, ERPs have the potential to become a routine adjunctive tool due to their low cost, high task controllability, and ease of repeated measurements. However, their clinical application still needs to address key standardization issues, including unifying ERP task parameters, electrode layouts, and analysis pipelines, as well as completing validation of reliability and validity in multi-center samples. The establishment and standardization of a multimodal ERP framework are expected to advance AD neurophysiological markers from theory to clinical practice.

## 3 Electroencephalography

### 3.1 Power spectrum features and early changes

Resting-state electroencephalogram (EEG) is a key non-invasive method for studying the neurophysiological abnormalities of AD. Among its many electrophysiological indicators, a well-established feature is that the EEG rhythms of AD patients exhibit a global slowing relative to healthy peers. This is characterized by a shift of the EEG power spectrum toward lower frequencies, manifesting as an increase in power in the slow-wave delta (approx. 1–4 Hz) and theta (approx. 4–8 Hz) bands, with a corresponding decrease in power in the fast-wave alpha (approx. 8–12 Hz) and beta (approx. 13–30 Hz) bands (39, 40). Furthermore, the occipital dominant rhythm in AD patients, typically the alpha wave, also shows a decrease in its peak frequency, representing a slowing of the brain’s fastest spontaneous oscillatory frequency.

Importantly, this phenomenon is a dynamically progressive process and is not exclusively present in the AD stage. Research by Shim et al. has confirmed that this change is already evident in the MCI stage and can even be traced back to the earlier preclinical phase (41, 42). A study by López-Sanz et al. demonstrated that, starting from the Subjective Cognitive Decline (SCD) stage, patients already exhibit reduced occipital alpha power compared to healthy peers, accompanied by disruptions in alpha-band network synchronization. This alpha-band dysregulation persists through the SCD, MCI, and AD stages, indicating that the slowing of brain electrical frequencies progressively worsens with disease advancement (43). To more precisely understand the neurophysiological basis of this phenomenon, Donoghue et al. (44) began to decompose the EEG power spectrum into periodic oscillatory activity and aperiodic 1/f background activity. This method can distinguish that the observed power changes are mainly caused by the attenuation of periodic rhythms such as alpha and beta, rather than by alterations in the brain’s background signal.

To translate these complex spectral changes into clinically applicable quantitative metrics, researchers often employ the ratio of high-frequency power to low-frequency power (e.g., the (α+β)/(θ+δ) spectral ratio) as a global assessment tool. A study by Kopčanová et al. confirmed that this spectral ratio is significantly reduced in AD patients compared to healthy controls, suggesting that this metric can serve as a sensitive quantitative parameter for the early detection of AD-related functional changes and for dynamically monitoring cognitive decline (45). In summary, EEG power spectrum abnormalities are not only an important signal for the early identification of AD but also provide a foundation for understanding its neurophysiological mechanisms.

### 3.2 Abnormalities in brain network functional connectivity

In addition to abnormalities in EEG frequency components, the connectivity patterns of the brain’s functional networks are also significantly altered in AD patients. By analyzing the degree of synchronization of brainwaves across different regions, Besthorn et al. found a general decrease in neural synchronization between different brain areas in AD patients. This phenomenon is evident in multiple frequency bands, including theta, alpha, and beta, reflecting an impairment in the functional integration capacity among various cortical regions (46). Furthermore, recent studies have applied a mathematical approach known as “Graph theory” to analyze the overall network structure. For instance, a study by Franciotti et al. discovered that in the early stages of AD, the brain network already begins to lose its efficient “small-world” properties. This indicates that as synaptic connections are damaged, this highly efficient system gradually degenerates, with pathways responsible for long-distance communication becoming weaker, causing the brain network to evolve into a more randomized and fragmented structure (47, 48).

However, against this backdrop of generalized connectivity impairment, a study by Oikonomou et al. found that some AD patients exhibit abnormal hypersynchronization in the theta band. This phenomenon does not represent a restoration of function but is more likely a local, inefficient compensatory mechanism. Mechanistically, as precise high-frequency networks degenerate, the resulting cortical disinhibition triggers pathological low-frequency hyper-coupling that lacks temporal precision and induces signal interference (49). After the brain’s efficient alpha-band communication network is damaged, some remaining neural circuits may attempt to maintain information transfer through low-frequency oscillations. However, this compensation is pathological and may, in fact, exacerbate the disarray of the overall network function (50).

The combined analysis of EEG spectral and network features also has value in differential diagnosis. For example, a study by Simfukwe et al. found that MCI patients of the AD type had significantly lower posterior alpha power than MCI patients with dementia with Lewy bodies, while their brain network connectivity also showed different patterns of change. These results suggest that different types of dementia may have specific electrophysiological signatures, providing potential biomarkers for disease subtyping (51). In summary, these findings at the brain network level not only deepen our understanding of the pathophysiology of AD but also provide important theoretical and technical support for the future development of early diagnosis, disease classification, and personalized intervention strategies.

### 3.3 Nonlinear analysis and clinical application prospects

The slowing of EEG rhythms and the disconnection of brain functional networks reflect a deeper change: the overall function of the brain as a complex dynamical system is degrading. This degradation can be quantified through nonlinear dynamics analysis, whose core metric is the complexity of the EEG signal (52). These methods, such as entropy and fractal dimension, aim to assess the richness, regularity, and predictability of the signal, thereby providing a higher-dimensional perspective for understanding the decline in the brain’s information processing capacity in AD. A study by Mizuno et al. found that, compared to a healthy control group, the EEG signal complexity in AD and MCI patients was significantly reduced. This indicates that the brain’s electrical activity becomes more monotonous, regular, and predictable, signifying a transition of the brain from a healthy, highly adaptive complex state to a more stereotyped and restricted pathological state (48, 53). Concurrently, artificial intelligence techniques such as machine learning have been applied to EEG data analysis, significantly improving the accuracy of early AD identification and the prediction of MCI progression to AD, offering new avenues for non-invasive detection and personalized intervention (54).

Longitudinal cohort studies have confirmed that certain baseline EEG abnormalities (such as elevated theta power and network connectivity impairments) can effectively predict the future rate of cognitive decline in high-risk populations. For example, a study by Jelic et al. found that MCI patients with higher baseline theta power were more likely to experience further deterioration in cognitive function during follow-up, eventually progressing to AD. This has significant implications for risk assessment and early intervention (55). In conclusion, with the popularization of portable EEG devices and the continuous optimization of artificial intelligence algorithms, EEG-based biomarkers are poised to develop into a scalable, low-cost clinical tool. It can not only be applied to early large-scale screening in resource-limited environments but also serve as an objective efficacy monitoring indicator in clinical trials for new drugs, providing key support for achieving precise diagnosis and personalized intervention for AD.

## 4 Transcranial magnetic stimulation-electroencephalogram

### 4.1 TMS-EEG reveals neurophysiological features of AD

Transcranial magnetic stimulation (TMS) delivers magnetic pulses to induce neurophysiological activity in the cerebral cortex. When combined with high-temporal-resolution electroencephalography (EEG), it allows for the precise and accurate recording of brain electrical activity. This non-invasive TMS-EEG method provides direct information about cortical reactivity and inter-regional neuronal connectivity (56). Current research primarily investigates the neurophysiological activity induced by TMS-EEG in the prefrontal cortex (PC), left dorsolateral prefrontal cortex (DLPFC), and primary motor cortex in individuals with AD, focusing on cortical excitability, neuroplasticity, and oscillatory dynamics. Casula et al. (57) applied TMS-EEG to induce neural activity in the PC, the DLPFC, and the left posterior parietal cortex (PPC) to explore cortical excitability levels. Their findings confirmed regional hyperexcitability in the PC of AD patients, and this cortical excitability level correlated with the degree of cognitive impairment and the levels of CSF pathological biomarkers. The precuneus is a critical component of the default mode network. Maiella et al. (58) simultaneously explored local cortical excitability and functional connectivity of the DMN in AD patients. Using neuronavigated TMS-EEG, they found cortical hyperexcitability in the precuneus of AD patients compared to healthy volunteers. However, this did not translate into effective propagation of the induced neural signal within the DMN. This DMN functional connectivity impairment is related to the severity of cognitive deficits in AD patients. This study further indicates that the combination of DMN functional connectivity impairment and increased cortical excitability constitutes a characteristic imaging feature in AD patients, providing important clues for understanding the neural mechanisms of AD.

A longitudinal 24-week follow-up study of Casula et al. demonstrated a significant reduction in frontal gamma oscillations in AD patients, which correlates positively with cortical plasticity impairment and serves as a predictive biomarker for subsequent cognitive decline (59). This suggests that TMS-EEG measurement of gamma activity could become an emerging method for assessing the progression of AD and synaptic dysfunction. A study on TMS-EEG biomarkers by Ferreri et al. (60) supports this view, showing that compared to healthy individuals, patients with amnestic mild cognitive impairment (aMCI) and AD have reduced beta and gamma inter-trial coherence (ITC), reflecting specific pathophysiological changes of AD. Lorenzo et al. (61) found that patients with MCI, prodromal AD, and AD with significant dementia all exhibited a marked loss of LTP-like cortical plasticity. They proposed that LTP-like cortical plasticity could be a predictor for the clinical progression to dementia in patients with prodromal memory impairment. Furthermore, LTP-like cortical plasticity might also be a potential neural mechanism for the cognitive improvements seen after rTMS treatment in AD patients. Li et al. (62) observed significant cognitive improvement in AD patients after six weeks of 20 Hz rTMS applied to the left DLPFC. Concurrently, long-term potentiation (LTP)-like cortical plasticity was significantly enhanced and positively correlated with the post-treatment cognitive improvement.

### 4.2 Clinical value of TMS-EEG in the diagnosis and treatment of AD

As a non-invasive neurophysiological assessment tool, TMS-EEG technology has shown practical value in the clinical diagnosis of AD. By quantifying TMS-induced electrophysiological signatures, this technique provides a neurophysiological basis for the early identification and differential diagnosis of AD. A study by Tăuţan et al. (63) demonstrated that by collecting over 150 time-domain features generated during TMS stimulation of the L-DLPFC and applying a random forest (RF) classifier algorithm, they could effectively distinguish between AD patients and healthy controls, achieving a best classification accuracy score of 92.95%. In the future, the integration of TMS-EEG with machine learning and deep learning methods will be instrumental for patient classification and longitudinal disease tracking. In addition, TMS-EEG can measure cortical plasticity and neural oscillations in AD patients, making it a valuable tool for evaluating the efficacy of therapeutic interventions and their underlying neural mechanisms. Lin et al. (64) proposed a novel accelerated high-dose intermittent theta-burst stimulation (iTBS) protocol applied to the left DLPFC of patients. This protocol significantly increased cortical excitability and neural oscillations, thereby validating its preliminary efficacy, safety, and tolerability for AD. In a randomized, phase 2, sham-controlled, double-blind trial, Koch et al. (65) used TMS-EEG to monitor changes in cortical activity and suggested that 24 weeks of repetitive magnetic stimulation of the precuneus may have a beneficial effect on cognitive and functional impairments in AD patients.

Due to its unique high spatiotemporal resolution and its ability to directly assess neural excitability and cortico-cortical functional connectivity, the TMS-EEG method has been increasingly applied in the field of AD research. However, future studies require larger cohort sizes and extended follow-up periods to further validate its accuracy and efficacy. Integration with neuroimaging is also necessary to expand the application of multimodal neurobiological markers in understanding neural mechanisms and clinical outcomes. A summary of parameters used in these studies is provided in **Table 3**.

**Table 3:** Summary of parameters in TMS-EEG studies of AD.

Reference	Stimulation area	Stimulation type	Stimulation method	Main electrophysiological findings	Functional/pathological interpretation
(57)	L-DLPFC, L-PPC, and PC	Single-pulse TMS	120 pulses, 2-4s, 90% adjRMT	Regional hyperexcitability in the precuneus (PC)	Correlates with the degree of cognitive impairment and CSF biomarker levels.
(59)	L-DLPFC, L-PPC, and PC	Single-pulse TMS	120 pulses, 2-4s, 90% adjRMT	Decreased frontal gamma oscillations	Reflects impaired cortical plasticity and serves as a predictor for subsequent cognitive decline.
(60)	Right first dorsal interosseous hotspot	Single-pulse TMS	100 pulses, 6-8s, 120% RMT	Reduced beta and gamma inter-trial coherence (ITC)	Reflects specific pathophysiological changes of AD.
(58)	L-DLPFC and PC	Single-pulse TMS	80 pulses, 2s, 100% RMT	Breakdown of induced signal propagation within the DMN	Indicates that local hyperexcitability fails to translate into effective network communication.
(62)	M1	High-frequency repetitive TMS	50 pulses, 15s, 90% RMT	LTP-like cortical plasticity was significantly enhanced	Positively correlated with post-treatment cognitive improvement.
(63)	L-DLPFC	Single-pulse TMS	120 pulses, 2-4s, 90% adjMT	Extraction of over 150 time-domain features	Achieved 92.95% accuracy in distinguishing AD from controls using RF classifier.
(64)	L-DLPFC	Single-pulse TMS	200 pulses, 4s, 80% RMT	Increased cortical excitability and neural oscillations	Validated preliminary efficacy, safety, and tolerability of iTBS protocol for AD.
(65)	PC	Single-pulse TMS	N/A, N/A, 100% adjRMT	Monitored changes in cortical activity	Suggested beneficial effect on cognitive and functional impairments in AD.

L-DLPFC, Left dorsolateral prefrontal cortex; L-PPC, Left posterior parietal cortex; PC, Precuneus; M1, Primary motor cortex; adjRMT, Adjusted resting motor threshold; RMT, Resting motor threshold; N/A, Not available in the original text.

## 5 Functional magnetic resonance imaging

Functional magnetic resonance imaging (fMRI) is a noninvasive magnetic resonance imaging method that capture functional changes in the brain during stimulation or lesions, with high temporal resolution superior to PET imaging, as well as spatial high resolution superior to EEG.

### 5.1 Resting-state functional magnetic resonance

#### 5.1.1 Resting-state functional magnetic resonance imaging reveals abnormal brain function in AD

Resting-state fMRI can map long-range, whole-brain functional connectivity networks based on the temporal coherence of slow (0.005–0.1 Hz) blood oxygen level-dependent (BOLD) activity (66). Agosta et al. (67) found that AD patients mainly exhibit changes in large-scale functional brain networks, not limited to the default mode network (DMN). Subsequent resting-state network studies had also demonstrated that these changes were associated with functional connectivity changes in the dorsal attention network (DAN), executive control network (ECN), and basal ganglia (68, 69). The analysis of functional connectivity in resting-state brain networks primarily involves three methods: seed-based connectivity analysis, graph theory analysis, and independent component analysis. Xiong et al. (69) conducted seed-based connectivity analysis and graph theory analysis using functional connectivity density values, functional connectivity strength, and graph theory index as analysis indicators. They identified changes in basal ganglia functional connectivity during cognitive decline, which may be related to Aβ deposition disrupting the NMDA receptor binding that maintains connectivity stability. Additionally, compared to stationary functional connectivity, dynamic functional connectivity exhibits unique time-series specificity. Gu et al. (70) utilized sliding window methods and k-means algorithms to reveal that AD may exhibit dynamic functional connectivity states and regional temporal variability that significantly distinguish it from healthy individuals, providing a new perspective for exploring the pathological progression of AD.

Functional connectivity primarily analyzes the correlations between the functions of different brain regions, while amplitude of low-frequency fluctuations (ALFF) and regional homogeneity (ReHo) are used to measure the spontaneous brain activity information of each brain region through rs-state fMRI. ALFF typically analyzes low-frequency signals in the range of 0.01–0.08 Hz, reflecting energy fluctuations in local brain regions within the low-frequency range (71). Wang et al. (72) found that abnormal ALFF features in AD and aMCI primarily manifested in the DMN. Most regions exhibited significantly reduced ALFF values, while hippocampal activity showed compensatory enhancement. These abnormalities correlated significantly with cognitive function. She et al. (73) found that, compared with healthy individuals, AD patients exhibited abnormal ReHo in the DMN brain region, further confirming that DMN disruption is a typical manifestation of AD. ALFF combined with ReHo metrics provides a more comprehensive view of brain functional abnormalities in disease. Yue et al. (74) compared brain functional imaging data from 26 aMCI patients and 26 healthy controls, revealing abnormal ALFF and ReHo phenomena in multiple cognitive-related brain regions among aMCI patients. This suggests that aMCI is not a single brain dysfunction.

#### 5.1.2 Resting-state fMRI combined with machine learning and deep learning for AD classification

AD is a progressive neurodegenerative disease, and MCI is considered to be the preclinical stage of the disease between healthy aging and dementia (75). Existing research has primarily focused on the binary classification of MCI and AD, with a small number of studies proposing the use of machine learning methods and deep learning techniques for the multi-stage classification of AD. Sun et al. (76) proposed a deep learning method based on convolutional neural networks (CNN) and recurrent neural networks (LSTM) to efficiently distinguish between AD and healthy individuals using resting-state fMRI brain functional networks, and to distinguish between stable MCI and progressive MCI at multiple time points. Noh et al. (77) constructed a 3D-CNN-LSTM classification model that utilized 4D fMRI data with temporal and spatial features to distinguish between four stages of AD, achieving an accuracy rate of 96.43%. Sadeghi et al. (78) used a machine learning approach based on resting-state fMRI and clinical data to accurately distinguish between AD-MCI, frontotemporal dementia, and healthy individuals, with an average accuracy of 91.1%. Machine learning and deep learning have improved the accuracy of multi-stage classification of AD, which is of great significance for the early diagnosis of AD.

#### 5.1.3 The potential of resting-state fMRI in AD treatment monitoring

The main treatment methods for AD include drug therapy and non-drug therapy. Resting-state fMRI can not only serve as an early biomarker for AD but also monitor the efficacy of disease treatment through dynamic functional changes. Yang et al. (79) conducted an analysis based on the resting -state fMRI metric ALFF and found that donepezil had a significant effect on spontaneous neural activity in the frontal lobe of AD, which was associated with improvements in cognitive function. Abnormal functional brain connectivity in the DMN is one of the most prominent neuroimaging features in AD patients. In a randomized controlled trial, Musaeus et al. used resting-state fMRI to investigate whether a 16-week aerobic exercise intervention altered functional connectivity changes in the DMN of AD patients. The results showed that no significant functional connectivity changes induced by the exercise intervention were observed (80). Summary of parameters in resting-state fMRI studies in AD are shown in **Table 4**.

**Table 4:** Summary of parameters in resting-state fMRI studies of AD.

Reference	Data source	Analytical method	Main functional alterations	Functional/pathological interpretation
(69)	ADNI	seed-based connectivity analysis and graph theory analysis	Altered functional connectivity of basal ganglia	Correlates with cognitive decline; likely related to Aβ disrupting NMDA receptor binding.
(70)	ADNI-2	sliding‐window approach and the k‐means algorithm	Abnormal dynamic functional connectivity states	Reveals high temporal variability that significantly distinguishes AD from healthy individuals.
(72)	clinical	ALFF	Reduced ALFF in DMN; compensatory enhancement in hippocampus	Objective reflection of regional energy fluctuation abnormalities associated with cognitive scores.
(73)	clinical	ReHo and VMHC	Abnormal ReHo in the DMN brain region	Further confirms that DMN disruption is a typical manifestation of AD.
(74)	clinical	ALFF and ReHo	Abnormal ALFF and ReHo in multiple cognitive-related brain regions	Demonstrates that aMCI involves widespread, rather than single-region, brain dysfunction.
(76)	ADNI	a CNN combined LSTM algorithm	Differentiated AD/healthy and stable/progressive MCI	Highlighted the value of deep learning in multi-stage classification.
(77)	ADNI-2	a 3D-CNN-LSTM classification model	Extracted 4D fMRI temporal and spatial features	Achieved 96.43% accuracy in multi-stage AD classification.
(78)	ANDI and FTLDNI	XGBoost	Accurately distinguished AD-MCI, FTD, and healthy individuals	Average classification accuracy reached 91.1%.
(79)	clinical	ALFF	Donepezil significantly affected spontaneous neural activity in the frontal lobe	Associated with improvements in cognitive function.
(80)	clinical	seed-based analysis, ICA-based analysis	No significant functional connectivity changes induced by 16-week aerobic exercise	Suggests 16 weeks may be insufficient to alter DMN resting-state connectivity.

ADNI, Alzheimer’s Disease Neuroimaging Initiative; FTLDNI, Frontotemporal Lobar Degeneration Neuroimaging Initiative; ALFF, amplitude of low-frequency fluctuation; ReHo; regional homogeneity; VMHC, voxel-mirrored homotopic connectivity; CNN, convolution neural network; LSTM, multi-layer long short-term memory; XGBoost, gradient-boosted decision trees.

### 5.2 Task-based fMRI

#### 5.2.1 Task-based fMRI and neuropsychological assessments reveal underlying neural mechanisms

Task-based fMRI characterizes subjects’ different cognitive states during tasks by recording changes in neurogenic oxygenation concentrations induced by visual, auditory, or other stimulation tasks (17). Integrating task-based fMRI with neuropsychological assessments facilitates a comprehensive investigation into the neural mechanisms underlying cognitive deficits in AD patients. Different components of executive function exhibit developmental and degenerative changes across various age stages, such as persistent decline in working memory capacity and inhibitory control throughout adulthood and into old age (81). As the severity of cognitive impairment increases, patients with AD and MCI demonstrate more pronounced progressive deficits in executive functioning compared to healthy elderly individuals (82). Consequently, task-based fMRI studies investigating AD frequently employ paradigms related to executive functions, such as the N-back working memory task and the Stroop inhibitory control task.

Working memory impairment is a significant cognitive deficit characteristic observed in AD patients. The N-back task serves as a classical experimental paradigm for assessing working memory function. Lim et al. (83) found that Alzheimer’s patients exhibited lower accuracy in performing the 1-back task compared to healthy controls. Neuroimaging studies primarily revealed decreased activation in the prefrontal cortex and increased activation in parietal neural networks, indicating that abnormal activity in specific brain regions was closely associated with deficits in working memory. The Zhang et al. study found that patients with SCD may have sustained neurological damage during the N-back task, which mainly showed vascular and the metabolic abnormalities in the frontal brain regions, while the parietal lobe did not show any significant difference. These results suggested that patients experienced vascular and metabolic regulation disorders related to cognitive functions; the differences between the groups were even more pronounced with the cognitive load, suggesting that the high-loaded task is more revealing of the pathological features of SCD (84). By examining the neural activation patterns elicited by working memory tasks of varying difficulty levels, this approach may facilitate the assessment of different cognitive stages in AD.

The Stroop task is a widely used neuropsychological assessment for evaluating inhibitory control and conflict resolution, which, when combined with neuroimaging techniques, allows for systematic investigation of activation patterns across various brain regions involved in cognitive inhibition processes. In the study conducted by Li et al., nine MCI and ten AD patients performed the Stroop color-word paradigm during the fMRI scan. The results indicated that prefrontal cortex activity significantly differentiated MCI and AD patients from healthy controls. Compensatory activation was observed in the prefrontal cortex of MCI patients, whereas AD patients exhibited prefrontal cortex dysfunction, confirming that prefrontal cortical damage in AD contributes to cognitive deficits (85). A recent systematic review further demonstrates that the Stroop task can serve as a key tool for distinguishing between individuals with cognitive impairment and healthy individuals. This shift from compensatory activation in the prefrontal cortex to decompensatory hypactivation is of significant importance for understanding the disease progression of AD (86). The prefrontal cortex is the core neural basis of executive function (87). Its structure and integrity are critical for understanding cognitive decline in AD and represent the central focus of task-based fMRI imaging biomarkers. A summary of task-based fMRI studies in Alzheimer’s disease is presented in **Table 5**.

**Table 5:** Summary of parameters in task-based fMRI studies of Alzheimer’s disease.

Literature	Paradigms	Changing cortical region	Imaging technology	Main activation alterations	Functional/pathological interpretation
Lim et al., 2008 (83)	1-back	the left frontal pole, vlPFC, insula, PMC; left precuneus	fMRI	FP/vlPFC/insula/PMC↓; precuneus↑	Suggests specific regional neural recruitment deficits leading to lower working memory accuracy.
Zhang et al., 2021 (84)	N-back	the frontal lobe	fMRI	Vascular and metabolic abnormalities	High cognitive load tasks are more sensitive in revealing pathological features in SCD patients.
Li et al., 2009 (85)	stroop	ACC, PFC, IPL, insula	fMRI	frontoparietal network↓	PFC dysfunction

vIPFC, ventrolateral prefrontal cortex; PMC, premotor cortex; ACC, anterior cingulate cortex; PFC, prefrontal cortex; IPL, inferior parietal lobule.

#### 5.2.2 Clinical applications of task-based fMRI

Task-based fMRI, by capturing BOLD signal fluctuations during the performance of specific cognitive tasks, offers a crucial neuroimaging modality for elucidating the neural substrates underlying cognitive domain impairments in AD (88). Compared with resting-state fMRI, which focuses on intrinsic network activity, task-based fMRI can characterize brain functional integrity, efficiency, and compensatory ability in the context of cognitive challenges, thereby complementing the assessment of network dynamic responses (89). Therefore, task-based fMRI shows important clinical application potential in early diagnosis, disease progression prediction, and treatment effect evaluation (90).

Numerous studies suggest that AD-related functional abnormalities are not simply linear declines but rather follow a trajectory of “hyperactivation–hypoactivation” that changes as the disease progresses (91). In the very early stages of the disease (such as MCI or individuals who carry the APOE ϵ4 allele but remain cognitively normal), task-based fMRI often shows “compensatory hyperactivation,” which is commonly seen in the medial temporal lobe (especially the hippocampus) and prefrontal cortex, and is particularly prominent when subjects are still able to complete moderately difficult memory tasks (92). This phenomenon is understood as resource mobilization in the context of decreased efficiency: when pathological load affects neural processing efficiency at an early stage, individuals need to recruit more neural resources to maintain surface behavioral performance. However, longitudinal studies have shown that individuals who exhibit stronger hyperactivation at baseline actually experience faster cognitive decline in the future, suggesting that this compensation is a precursor to neural system dysfunction (91). As the disease progresses to the AD dementia stage, the brain’s activation pattern shifts to a pronounced “hypoactivation,” a phenomenon that is particularly significant in key memory network nodes such as the hippocampus and precuneus. This is considered a direct functional consequence of synaptic dysfunction and neuronal loss, reflecting the brain’s inability to effectively mobilize the necessary neural resources when faced with cognitive challenges (93).

Given that task-based fMRI can capture functional compensation in the early stages of disease, it has great potential for predicting the conversion of MCI to AD dementia. Miller et al. found that in cognitively normal older adults or MCI patients, those with stronger hyperactivation of the hippocampus or prefrontal cortex during baseline memory tasks tended to experience faster cognitive decline and a higher risk of AD conversion (94). Gordon et al. (95) found that in cognitively normal older adults, CSF tau and ptau levels were positively associated with task-evoked hyperactivation of attentional networks during Stroop and semantic judgment tasks. These functional changes preceded any behavioral impairment, indicating that task-based fMRI can detect AD-related neural abnormalities prior to clinical symptoms. In terms of predictive methods, the choice of task paradigm is crucial. Although impaired episodic memory is a hallmark of AD, MCI subjects show considerable variability in their performance on this task, which can interfere with the interpretation of differences in neural activation. Therefore, some studies have suggested that using relatively preserved semantic memory tasks (such as celebrity judgment) may be more advantageous (96). Such tasks are like standardized “stress tests.” Because task performance is more consistent across different subjects, the observed differences in brain activation more purely reflect the differences in neural efficiency caused by AD pathology, thereby more accurately predicting future cognitive decline (97). Furthermore, the integration of task-based fMRI with machine learning algorithms, combined with structural MRI and APOE genotyping biomarkers, holds promise for enhancing the accuracy and stability of personalized predictive models (98, 99).

As a non-invasive, repeatable objective imaging metric, task-based fMRI can also be used to assess the central effects and efficacy dynamics of drug and non-drug interventions (90). Several clinical studies on cholinesterase inhibitors have used task-based fMRI to explore their efficacy mechanisms (100). Research has found that after treatment, patients exhibit altered brain activation patterns when performing memory or attention tasks. For example, studies by Saykin et al. (101) and Bakker et al. (102) demonstrated that activation in task-related brain regions such as the medial prefrontal cortex and temporal lobe is enhanced, or activation levels in previously overactive regions such as the hippocampus decrease and normalize. These functional changes are often associated with improvements in cognitive abilities, providing objective evidence of the drug’s effects on the central nervous system. This facilitates early assessment of drug efficacy in clinical trials, thereby accelerating the development of new drugs (103). Biel et al. applied functional network maps that the task-based fMRI defined to individual Tau-PET images, enabling precise prediction of decline in specific cognitive domains and opening up new directions for personalized diagnosis and treatment of AD and clinical trial design (104).

## 6 Structural MRI

### 6.1 T1/T2

#### 6.1.1 Clinical features

**Figure 2:**
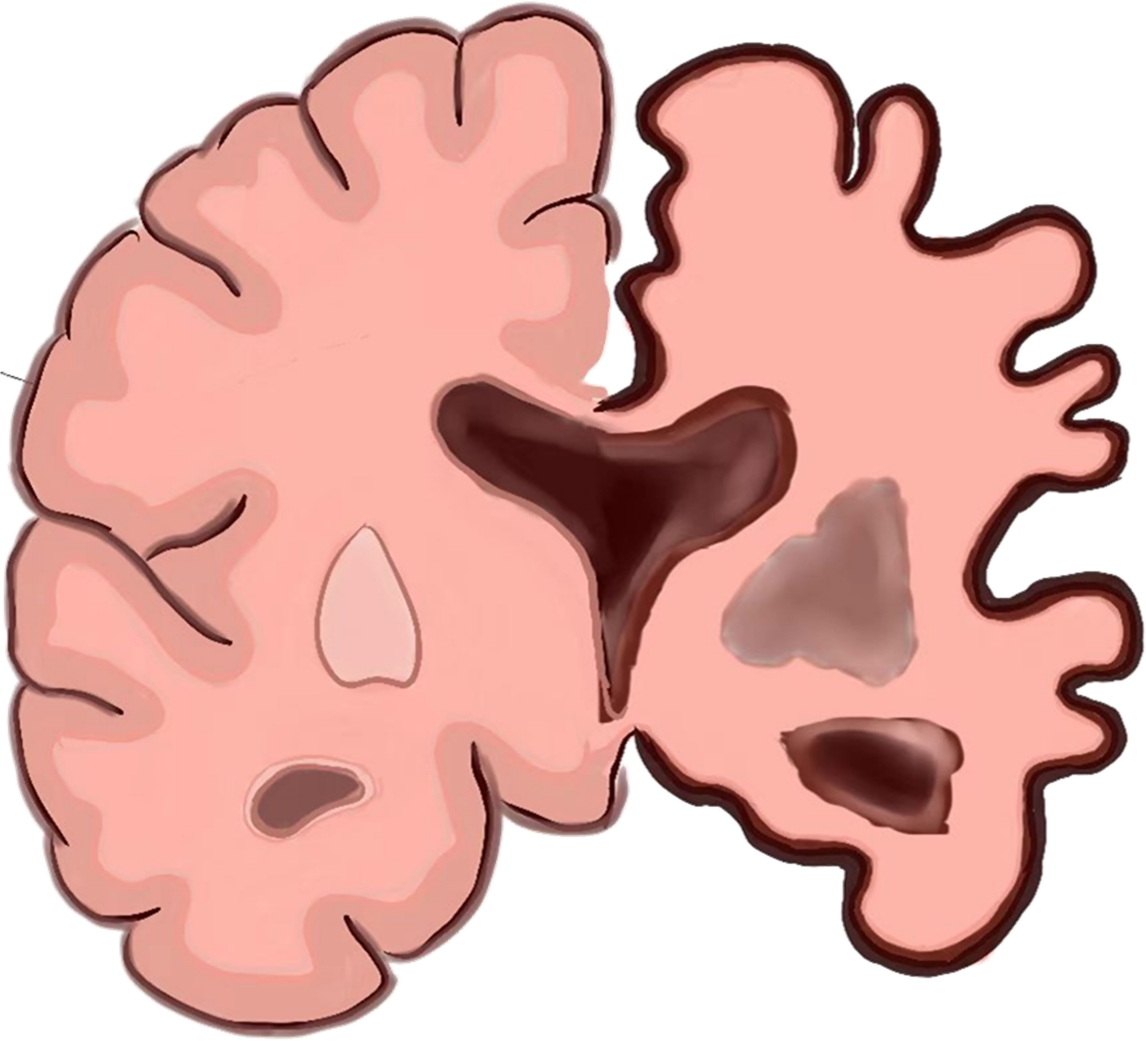
Schematic diagram of brain atrophy in AD compared to a normal control brain.

Medial temporal lobe atrophy observed on structural MRI is widely recognized as a neuroimaging hallmark of AD (105). In a study by Paola et al. (106) voxel-based morphological analysis was used to delineate the medial temporal lobe structures within the brain, demonstrating that cortical structural atrophy in the medial temporal lobe cortex, such as the hippocampus, parahippocampal gyrus, and entorhinal cortex, significantly impaired patients’ episodic memory. Feng et al. (107) refined the structure of the hippocampal subregions and found that the volumes of most hippocampal subregions in AD patients were significantly smaller than those in healthy controls and MCI patients, accompanied by more severe cognitive and orientation impairments. The entorhinal cortex is located in the anterior part of the parahippocampal gyrus and is closely related to the output of the hippocampus (106). Tran et al. (108) primarily observed cortical atrophy and functional inhibition in the lateral entorhinal cortex in aMCI. This imbalance in damage to the medial and lateral entorhinal cortex was consistent with the deposition sites of tau neurofibrillary tangles, a pathological feature of early AD. The amygdala is also one of the core brain regions of the medial temporal lobe and is associated with emotional dysfunction and memory impairment in AD patients. Wang et al. (109) found that the bilateral amygdala volume of AD patients was smaller than that of the control group, and there was a significant correlation between the amygdala and the hippocampus volume. **Figure 2** illustrates brain atrophy in AD compared to normal controls. The AD brain shows characteristic structural changes, including ventricular enlargement, hippocampal atrophy, and cortical thinning. A study on high-resolution T2 imaging further demonstrated that medial temporal lobe atrophy exhibits specific stage patterns along the AD continuum. And the atrophic brain regions correspond to different cognitive declines, providing imaging markers for monitoring stage-specific cognitive deterioration in the disease (110). Additionally, the T1-weighted to T2-weighted image intensity ratio was originally proposed by Glasser et al. (111) as a non-invasive approach to assess cortical myelin content. However, Pelkmans et al. (112) found that the T1/T2 ratio in gray matter is significantly elevated in AD patients, which is opposite to the phenomenon observed in demyelinating lesions. They suggested that this increased ratio may be driven by factors other than myelin content, and future studies should further explore its neurobiological significance.

#### 6.1.2 Clinical application

Structural MRI can quantify changes in the volume of human brain tissue. Currently, some studies focus on using multimodal imaging markers from structural MRI to accurately distinguish between healthy elderly people and MCI and AD. Zhang et al. (113) integrated two features, including gray matter (GM) volume and lateralization index (LI) of structural MRI, and applied multiple classification algorithms to construct a binary classification model for healthy elderly individuals, early mild cognitive impairment (EMCI), late mild cognitive impairment (LMCI), and AD patients. The results showed that, across all algorithm models, the classification accuracy for the healthy group and AD was the highest, with the highest accuracy reaching 98.0909%. Gupta et al. (114) extracted combined features of voxel-based morphometry, hippocampal volume, and cortical and subcortical segmented regions from T1-weighted structural MRI images to improve the accuracy of AD diagnosis and early diagnosis. In addition, frontotemporal dementia and AD share overlapping clinical manifestations. Yu et al. found through sMIR that the AD resemblance atrophy index based on brain atrophy was helpful in distinguishing between these two similar neurodegenerative diseases (115).

### 6.2 DTI

Diffusion tensor imaging is a magnetic resonance imaging technique based on the diffusion characteristics of water molecules in brain tissue, which can non-invasively quantify the microstructural integrity of white matter tracts (116). Commonly used diffusion tensor imaging metrics include fractional anisotropy (FA), mean diffusivity (MD), axial diffusivity (DA), and radial diffusivity (RD). They reflect the directionality and integrity of white matter tracts as well as the structural status of myelin and axons (117, 118). Patients with AD universally exhibit decreased FA alongside increased MD, DA, and RD in the white matter. This microstructural damage is widely present across the white matter tracts of multiple brain regions and correlates with the cognitive impairment and disease severity of AD (119).

Limbic system-related tracts, including the cingulum and fornix, are key regions affected in AD. The decreased fractional anisotropy in these regions correlates with tau protein deposition and precedes macroscopic white matter atrophy (120), suggesting that microstructural damage may serve as a direct imaging manifestation of early pathology in AD. A one-year longitudinal study also demonstrated that the AD group exhibited decreased FA and increased MD in the corpus callosum, cingulum, fornix, and widespread white matter tracts. Moreover, damage to the hippocampal cingulum demonstrated disease specificity as it was observed exclusively in the patient group (121). A two-year longitudinal study found that in patients progressing from MCI to AD, DA and RD values were significantly elevated in the corpus callosum, internal capsule, corona radiata, cingulum, superior longitudinal fasciculus, and fornix. Among these metrics, changes in DA were closely related to the decline in cognitive scores, indicating that DA is more sensitive to white matter microstructural damage during the progression of AD (122). Additionally, Raghavan et al. (123) further demonstrated that the fractional anisotropy of the genu of the corpus callosum could capture microstructural damage in white matter fibers and independently predict cognitive decline.

Classification studies based on support vector machines show that FA and MD metrics possess a strong capacity to distinguish AD from healthy control groups, reaching accuracies of 89% and 85% respectively. This further indicates that diffusion tensor imaging metrics combined with machine learning methods hold potential application value in the auxiliary diagnosis of AD (124). Diffusion tensor imaging combined with FA, MD, DA, and RD metrics can comprehensively reflect the spatial specificity of white matter microstructural damage in AD. This damage is tightly linked to tau pathology and cognitive decline. Furthermore, these microstructural changes can be detected earlier than the macroscopic atrophy observed on T1-weighted magnetic resonance imaging, thus providing a crucial imaging basis for early diagnosis and longitudinal monitoring.

The medial temporal lobe atrophy and white matter microstructural damage presented by structural magnetic resonance imaging provide a direct anatomical basis for the decreased functional connectivity observed in functional magnetic resonance imaging. **Table 6** summarizes the key findings of T1/T2 and DTI in AD research.

**Table 6:** Summary of T1/T2 and DTI findings in AD.

Reference	Modality	Key brain regions	Key metrics	Main structural alterations	Functional/pathological interpretation
(106–108)	T1	Medial temporal lobe (Hippocampus, entorhinal cortex, amygdala)	Volume/Cortical thickness	Significant volume reduction and cortical atrophy	Strongly associated with episodic memory impairment and tau tangle deposition sites.
(110)	T2	Medial temporal lobe	Volume	Stage-specific atrophy	Providing imaging markers for monitoring stage-specific cognitive deterioration
(119–121)	DTI	Whole-brain white matter, corpus callosum	FA, MD	Decreased FA, increased MD	Reflects widespread loss of myelin and axonal integrity; precedes macrostructural atrophy.
(122, 123)	DTI	White matter fiber tracts	DA, RD	Increased DA and RD	Independent and highly sensitive predictors of longitudinal cognitive decline in the MCI to AD transition.

## 7 Other advanced quantitative magnetic resonance imaging technologies

In addition to conventional structural and functional imaging, arterial spin labeling (ASL) and quantitative susceptibility mapping (QSM) provide new perspectives for the early recognition of AD.

### 7.1 Quantitative susceptibility mapping

Quantitative susceptibility mapping (QSM) is an advanced quantitative technique based on magnetic resonance phase information, capable of precisely measuring the spatial distribution of magnetic susceptibility within brain tissue, with extremely high sensitivity to paramagnetic substances such as iron. In AD, abnormal iron metabolism and oxidative stress are considered crucial driving factors of neurodegenerative lesions (125). Research by Merenstein et al. indicated that AD patients exhibit significant abnormal iron deposition in critical brain regions such as the hippocampus, caudate nucleus, putamen, and cortex (126). Localized iron overload detected using QSM technology has been proven to be closely related to the formation of amyloid-beta (Aβ) plaques and Tau protein tangles, and these two pathological proteins can bind iron ions and exacerbate neuronal toxic damage (127, 128). Clinical studies by Ayton et al. further pointed out that elevated QSM susceptibility in the temporal and parietal cortices is significantly positively correlated with the rate of cognitive decline and the risk of transition from MCI to AD (129). Therefore, the quantitative susceptibility metrics of QSM provide a unique *in vivo* imaging perspective for understanding the microscopic molecular pathology of AD and hold the promise of becoming an important imaging biomarker for monitoring early neural damage in the disease.

### 7.2 Arterial spin labeling

Arterial spin labeling (ASL) is a magnetic resonance perfusion imaging technique that quantifies cerebral blood flow (CBF) by magnetically labeling water molecules in arterial blood, and it has been applied to AD research due to its strong reproducibility and non-invasiveness. In AD, the deposition of Aβ in the walls of cerebral blood vessels can impair vascular endothelial function, subsequently leading to a reduction in regional cerebral blood flow (130). Alsop et al. (131) applied voxel-based analysis early on and found significant hypoperfusion in the temporal lobe, parietal lobe, frontal lobe, and posterior cingulate gyrus of AD patients. MCI patients exhibit hypoperfusion in posterior brain regions such as the precuneus, inferior parietal lobule, middle temporal gyrus, and occipital lobe, while hyperperfusion appears in the lentiform nucleus, possibly indicating that early MCI maintains cognitive function by increasing blood flow (132). Duan et al. (133) conducted a longitudinal ASL follow-up study encompassing healthy individuals, MCI, and AD populations, finding that stable MCI manifested as reduced CBF in the right temporoparietal region and right hippocampus, whereas progressive MCI showed widespread hypoperfusion in bilateral temporoparietal regions. Furthermore, baseline CBF in the posterior cingulate gyrus, superior medial frontal gyrus, and left inferior frontal gyrus can predict cognitive levels during the follow-up period. By detecting alterations in cerebral blood perfusion, ASL provides a vital basis for the early recognition of AD from the perspective of neurovascular function.

## 8 MEG

### 8.1 Basic principles and technical advantages of MEG

Magnetoencephalography (MEG) is a non-invasive neurophysiological technique that utilizes superconducting quantum interference devices (SQUIDs) to record the weak magnetic fields generated by neuronal currents in the brain. Unlike fMRI, which relies on blood oxygen level-dependent hemodynamic responses, MEG can directly measure neural activity. MEG not only possesses a millisecond-level temporal resolution comparable to EEG, but its magnetic field signals are also less affected by conductivity attenuation when penetrating the skull and scalp. Therefore, MEG exhibits higher spatial precision in cortical source localization (134). Simultaneously, MEG demonstrates extremely high sensitivity in detecting early neurophysiological abnormalities in patients with AD. Extensive resting-state MEG studies indicate that patients with AD and MCI exhibit neural oscillations characterized by enhanced low-frequency activity and reduced high-frequency activity, known as the slowing phenomenon (135, 136). Further multimodal research also supports this finding; for instance, Nakamura et al. (137) utilized PiB-PET and structural MRI to confirm underlying pathological status, demonstrating that resting-state MEG can detect specific signatures of abnormal neural activity during the MCI stage, particularly in the amyloid-positive preclinical AD phase. van Nifterick et al. (138) utilized source-reconstructed resting-state MEG technology to study cognitively normal carriers of autosomal dominant AD mutations (PSEN1/APP). The results revealed that even years before the expected onset, abnormal functional connectivity emerged in the hippocampus and core nodes of the default mode network, such as the precuneus and posterior cingulate gyrus. This abnormal phenomenon can be stably detected before the manifestation of macroscopic brain atrophy and cognitive decline, strongly supporting the hypothesis of the AD pathological progression where electrophysiological changes precede macroscopic anatomical damage.

### 8.2 Microscopic mechanisms of E-I imbalance, hypersynchronization, and spectral slowing

Although the abnormal increase in low-frequency and attenuation in high-frequency resting-state neural oscillations have been widely proposed in EEGstudies, MEG can analyze the anatomical origins of this phenomenon in finer spatial dimensions by relying on its cortical source localization technology (136). Current research generally suggests that in the early stages of AD, Aβ and Tau proteins exert distinct targeted toxicities, where Aβ tends to interfere with GABAergic interneurons, while Tau protein deposition specifically impairs excitatory neuron function. Both collectively trigger a local network excitation-inhibition (E-I) imbalance (139, 140). To quantify this microscopic pathology, Ranasinghe et al. combined macroscopic MEG spectral features with neural mass modeling to successfully map abnormal electroencephalographic oscillations to underlying neural microcircuit changes *in vivo*. This study confirmed that it is precisely this underlying E-I imbalance that directly leads to macroscopic electroencephalographic spectral changes, such as Aβ-induced low-frequency slowing and Tau-mediated high-frequency attenuation (139).

At the macroscopic brain network topological level, this local synaptic dysfunction does not immediately manifest as global network disconnection, but rather exhibits a complex non-linear temporal dynamic evolution. The study by Nakamura et al. showed that in cognitively normal (CN) individuals positive for Aβ deposition, medial prefrontal regions (such as the medial frontal cortex and anterior cingulate cortex) exhibit significant alpha-band EEG power enhancement and hypersynchronization features (137). This early hyperconnectivity may not merely reflect a compensatory mechanism, but rather a direct projection of neuronal hyperexcitability resulting from the aforementioned underlying E-I imbalance at the whole-brain network level (139, 140). More importantly, this abnormal synchronization possesses high specificity and continuity in both the frequency domain and spatial distribution. For example, the study by Lopez et al. (141) demonstrated that patients with progressive mild cognitive impairment (pMCI) specifically exhibit a significant enhancement of functional connectivity in the alpha band during the early stages of the disease, known as hypersynchronization. From the perspective of network topology, this hypersynchronization is highly concentrated in the anterior cingulate cortex (ACC) and temporo-occipital regions. The network hyperactivation in these specific frequency bands and brain regions not only reflects the continuous pathological remodeling of macroscopic brain networks by underlying neuronal hyperexcitability, but also serves as a highly sensitive neuroelectrophysiological biomarker predicting the transition from MCI to AD.

However, as the disease irreversibly progresses toward the dementia stage of AD, the long-term hyperexcitability of neurons and the continuous accumulation of synaptic toxicity ultimately drive the brain network toward a decompensation phase, with the MEG connectivity profile rapidly transitioning from early hypersynchronization to widespread long-range disconnection. Graph theoretical analysis by Engels et al. indicated that the network centrality of the precuneus and posterior cingulate gyrus, core nodes of the posterior default mode network, significantly decreases, and damage to these critical nodes hinders long-range communication within large-scale brain networks, ultimately leading to a substantial reduction in whole-brain information integration efficiency (142). This phenomenon is consistent with the hyperactivation to hypoactivation trajectory demonstrated by task-based functional magnetic resonance imaging. Both techniques capture the dynamic evolution from the compensatory stage to network collapse within the AD spectrum, highlighting the significant role of electrophysiological and hemodynamic indicators in tracking disease progression.

### 8.3 Clinical translation potential of MEG and AI-assisted diagnosis

From the perspective of clinical translation, MEG-derived features demonstrate high biomarker value in assessing AD disease progression and cognitive impairment risk. A cross-sectional analysis by Wiesman et al. (135) confirmed that the pattern of neural spectral slowing with high spatial specificity can not only accurately reflect the degree of cognitive impairment in specific domains such as attention, processing speed, and language function, but is also closely related to the local Aβ pathological burden in the brain. Faced with the high-dimensional and non-linear complex spatiotemporal features of MEG signals, traditional statistical analyses often struggle to comprehensively mine their deep information. In recent years, the BioFIND multicenter resting-state MEG large database constructed by Vaghari et al. (143) effectively broke the sample size bottleneck faced by AI algorithms. This study aggregated high-quality data from 324 patients with MCI and healthy controls, providing ample material for extracting high-dimensional brain features via machine learning. This work laid the foundation for verifying the cross-center generalization ability of classification models and promoted the integration of MEG-derived features with artificial intelligence technology. In addition, researchers utilized beamformer algorithms such as linearly constrained minimum variance (LCMV) to achieve precise source localization, and combined deep learning models to automatically extract neurophysiological indicators. For instance, Lopez-Martin et al. (144) adopted a randomized convolutional neural network architecture to significantly improve recognition performance in early AD stages by automatically inducing deep features within MEG signals. Meanwhile, the Deep-MEG framework proposed by Giovannetti et al. (145) achieved a classification accuracy approaching 90% in predicting early signs of AD by integrating multi-band functional connectivity metrics with spatiotemporal convolutional neural networks. These studies provide an objective basis for AI combined with source-space electrophysiological data for precise AD staging and demonstrate significant clinical translation potential. In conclusion, supported by modern data and AI algorithms, MEG is poised to become a multidimensional, non-invasive prognostic biomarker with highly promising application prospects in the early recognition and disease monitoring of AD. The parameters of the MEG in the Alzheimer’s study are summarized in **Table 7**.

**Table 7:** Summary of representative MEG studies across the AD.

Reference	Population	Key metric/paradigm	Main alterations and brain regions	Functional/pathological interpretation
(137)	CN (Aβ+), MCI	Multimodal integration (MEG, PiB-PET, structural MRI)	Increased alpha power and hyper-synchronization in medial PFC and ACC in prodromal stages.	Reflects early network hyperexcitability driven by an underlying E-I imbalance.
(138)	PSEN1/APP carriers	Source-reconstructed resting-state MEG	Functional connectivity abnormalities in the hippocampus and DMN core nodes detected years before clinical symptoms.	Early electrophysiological changes precede macroscopic anatomical atrophy by years.
(139)	AD continuum	Spectral features and Neural mass modeling	E-I imbalance characterized by Aβ-mediated low-frequency slowing and Tau-mediated high-frequency impairment.	Directly maps macroscopic spectral slowing to microscopic excitatory-inhibitory neuronal dysfunction.
(141)	pMCI	Resting-state MEG	Enhanced alpha-band hyper-synchronization in the ACC and temporo-occipital networks.	Highly sensitive neurophysiological biomarker predicting the transition from MCI to AD.
(135)	AD continuum	Cross-sectional spectral analysis	Spatially resolved spectral slowing positively associated with local Aβ burden and domain-specific cognitive decline.	Spectral slowing pattern acts as an accurate proxy for cognitive impairment severity.
(144, 145)	MCI and Mild AD	MEG and Deep Learning (CNN)	Integration of spatiotemporal features achieving high classification accuracy for early-stage AD.	Demonstrates significant clinical translation potential for AI-assisted precise staging.

CN, Cognitively Normal; MCI, Mild Cognitive Impairment; pMCI, Progressive MCI; PFC, Prefrontal Cortex; ACC, Anterior Cingulate Cortex; PCC, Posterior Cingulate Cortex; DMN, Default Mode Network; E-I, Excitation-Inhibition.

## 9 Functional near-infrared spectroscopy

Functional near-infrared spectroscopy (fNIRS) is a non-invasive brain functional imaging technology based on neurovascular coupling mechanisms. When local neuronal activity increases, the resulting metabolic demand triggers microvascular dilation and an increase in blood flow, manifesting as relative changes in the concentrations of oxygenated hemoglobin (HbO) and deoxygenated hemoglobin (HbR). fNIRS utilizes near-infrared light to penetrate the skull and capture hemodynamic responses in real time, thereby indirectly evaluating the level of neural activity in the cerebral cortex. Compared to fMRI, fNIRS possesses advantages in temporal resolution, resistance to motion artifacts, and portability.

### 9.1 Regional hemodynamics and task-state activation

The most fundamental measurement metrics of fNIRS are task-induced concentration changes of HbO and HbR, reflecting the activation intensity of the local cortex. Most studies primarily demonstrate weakened activation intensity in the prefrontal, parietal, and occipital lobes in AD. As early as 1997, Fallgatter et al. (146) and others found that AD patients lose left-hemispheric dominance during the verbal fluency test (VFT), presenting symmetrical bilateral prefrontal activation. Recently, yang et al. (147) further discovered decreased changes in HbO concentration and cortical activation in multiple prefrontal brain regions of AD patients when performing tasks. In addition to the VFT, numerous fNIRS studies have gradually expanded to memory tasks, such as the n-back working memory task (148), the visual memory span task (149), and the delayed matching to sample task (150). Liu et al. (150) utilized a delayed matching to sample (DMTS) task to reveal abnormal hemodynamic changes in the prefrontal, parietal, and occipital regions of aMCI patients. These findings are consistent with task-related hemodynamic signal decreases observed in specific brain regions using fMRI. Together, these results indicate that prefrontal hemodynamics is a specific biomarker for the early identification of AD.

### 9.2 Functional connectivity

Functional connectivity reflects the temporal correlation of neural activity between different brain regions and is a core brain network metric for maintaining cognitive function. Functional connectivity analyses using fNIRS and fMRI are both based on temporal signal correlations. Due to the spatial resolution limitations of fNIRS, its functional connectivity analyses primarily focus on local cortical networks. In contrast, fMRI studies of functional connectivity have increasingly identified connectivity disruptions in whole-brain networks associated with AD, such as the DMN, the dorsal attention network, and the executive control network. In recent studies on functional connectivity are scarce, and the results show discrepancies. Nguyen et al. (151) found enhanced right prefrontal and interhemispheric functional connectivity in MCI patients during resting states, while left and interhemispheric connectivity significantly decreased under verbal fluency tasks; this phenomenon may reflect insufficient neural compensation when AD patients execute cognitive tasks with high loads. Conversely, Chen et al. (152) discovered that as the severity of AD increases, the strength of prefrontal functional connectivity in patients exhibits a progressive declining trend from healthy individuals to MCI and then to AD. Whole-brain functional connectivity analysis by Zhang et al. (153) indicated a decline in brain network connectivity in MCI patients. Furthermore, the classification model they constructed showed that impaired long-range connections from the prefrontal to the occipital and parietal lobes are highly prominent and can serve as classification indicators between MCI and healthy control groups. Aside from undirected functional connectivity, a resting-state fNIRS study based on effective functional connectivity revealed that compared to healthy elderly individuals, MCI patients exhibit reduced coupling strength in multiple effective connection pathways between brain regions, mostly located between the bilateral prefrontal and occipital lobes (154). Existing evidence suggests that reduced prefrontal functional connectivity is a characteristic of fNIRS for the specific recognition of the AD population.

### 9.3 Signal complexity

Signal complexity can serve as a novel indicator for evaluating cognitive impairment in AD, primarily involving analyses such as multiscale entropy, spectral entropy, and sample entropy (SampEn). Li et al. (155) employed multiscale entropy to analyze resting-state fNIRS signals and found that the signal complexity of the default mode network, frontoparietal network, and attention network in AD patients was significantly lower than that in healthy controls, which may be related to neural network degeneration and a decline in information processing capacity. Research by Ferdinando et al. (156) showed that AD exhibits higher spectral entropy in the very low frequency (VLF) band compared to normal control groups. Since the VLF band reflects vasomotor activity, the aforementioned phenomenon implies that patients with AD suffer from pronounced vasomotor dysfunction, potentially related to Aβ deposition in blood vessels. Perpetuini et al. (157) discovered through sample entropy and multiscale entropy that complexity changes in abnormal prefrontal signals are also present in task-state studies of AD patients, holding potential for distinguishing healthy individuals from AD. Currently, no unified conclusion has been reached regarding changes in entropy metrics, which may be limited by various factors such as measurement states, task paradigms, and metric heterogeneity.

fNIRS can offer potential possibilities for the early recognition of AD from three dimensions, regional hemodynamics, functional connectivity, and signal complexity. The diminished task-related activation and decreased functional connectivity in the prefrontal cortex are spatially similar to the functional abnormalities of networks such as the DMN and ECN observed in resting-state fMRI. This demonstrates that the prefrontal cortex is one of the key brain regions susceptible to damage in AD. Furthermore, the reduced signal complexity in fNIRS cross-validates with the decreased multiscale entropy in the nonlinear analysis of EEG. This indicates that the information processing richness of the brain in AD undergoes degradation from both the perspective of hemoglobin concentration and neural electrical activity. However, as an emerging technology, fNIRS still requires longitudinal clinical studies and an exploration of its application value in monitoring pharmacological and non-pharmacological interventions.

## 10 Application of multimodal imaging technologies

In terms of clinical translation, multimodal metrics also contribute significantly. Combining ERP with other clinical or electrophysiological indicators can substantially improve predictive efficacy. Research by Doan et al. (158) showed that models built on prefrontal EEG and selective attention ERP can better predict the transition from MCI to AD, and concurrent integration with MMSE scores can further improve screening effectiveness. The combined application of structural magnetic resonance features with other imaging technologies such as PET, DTI, and fMRI can achieve superior diagnostic performance. For example, in a clinical study, Wu et al. (159) combined functional metrics from resting-state fMRI and structural metrics from structural magnetic resonance to demonstrate that multimodal changes in the DAN provide stable diagnosis of early AD progression. The integration of proteomics and imaging offers precision for targeted therapy. Afxenti et al. (160) combined proteomics and structural magnetic resonance to identify five core proteins, APP, VGF, APOE, SCG3, and NCAN, associated with the neuroimaging features of AD.

Existing research is increasingly focusing on the critical role of structural and functional connectivity coupling between DTI and fMRI (rsfMRI-FC/DTI-SC) in the progression of AD. Xu et al. (160) pointed out that the whole-brain SC-FC coupling strength gradually increases with AD disease progression and correlates negatively with MMSE scores, suggesting that changes in network coupling might be the cause of exacerbated cognitive impairment in AD patients. However, research by Sun et al. (161) presents a different perspective, showing that compared to healthy populations, the FC-SC coupling strength in MCI patients is significantly reduced, reflecting that the disruption of brain network synchronization may have already occurred in the early stages of the disease. The pathological stages and the direction of coupling changes may exhibit stage specificity. In Aβ-positive MCI patients, Aβ pathological deposition is negatively correlated with structural-functional coupling, while in Aβ-positive AD patients, this relationship reverses and becomes a positive correlation (162). The aforementioned study results suggest that coupling changes may not be a linear global process and require further revelation of pathological mechanisms through brain network modularity. Research by Piao et al. (163) demonstrated that the SC-FC coupling of MCI exhibits abnormal differences at the module level and is significantly correlated with network efficiency, providing a new perspective for exploring SC-FC as a potential imaging biomarker in the clinical practice of the disease.

Simultaneous EEG-fMRI technology quantifies the degree of synchronous association between neural oscillations and blood oxygen level-dependent signals. Impaired thalamic alpha-BOLD signal coupling may be a neurophysiological feature reflecting AD. Brueggen et al. (164) first applied this technology to AD research, finding that the patient group displayed a weakened positive correlation between alpha-band signal power and thalamic BOLD signals in the default mode network and thalamus, consistent with the pathological features of thalamocortical loop impairment. Michels et al. (165) further confirmed that this change is already present in patients with MCI. Furthermore, the pathological feature of cerebral amyloid deposition is linked to abnormal EEG-fMRI coupling at the hippocampus. Research on simultaneous electroencephalography and functional magnetic resonance in the field of AD is not yet complete, and future efforts need to further expand empirical research to validate its clinical value.

## 11 Discussion

AD, as a neurodegenerative disease, has become a major global public health challenge due to its increasing incidence, prevalence, and mortality rates. Identifying imaging biomarkers for the early stages of AD and for its differential diagnosis is of great significance for improving the diagnosis and treatment of this disease. The neurophysiological and neuroimaging techniques discussed in this paper reveal underlying pathological mechanisms of AD, demonstrating distinctive electrophysiological and imaging abnormalities. Additionally, by integrating these methods with machine learning models or deep learning algorithms, new possibilities for differentiating disease stages have been achieved.

However, these technologies may face different methodological challenges in AD research: (1) Compared with other imaging methods, ERP and EEG have significant temporal resolution, capturing the temporal characteristics of brain neural activity in detail, but their spatial resolution is low, making it difficult to accurately obtain the fine structure of the brain. (2) The brain electrical activity generated by TMS is affected by confounding factors such as auditory and somatosensory stimuli, which cause similar nonspecific scalp responses. Correct technical methods must be used to reduce the interference of these factors (166). (3) Resting-state functional data images are primarily derived from standardized imaging data provided by the AD Neuroimaging Initiative (ADNI), but the sample size is limited. Researchers may consider incorporating a larger number of clinical participants in future studies to complete clinical and cognitive assessments. (4) When combining deep learning and machine learning methods to distinguish and predict different stages of AD, it is feasible to explore the use of multimodal imaging data and clinical participant data to construct classification frameworks, thereby enhancing classification accuracy and generalizability. (5) Brain structural MRI segmentation is mainly divided into manual segmentation and automatic segmentation. Manual segmentation is highly dependent on the professional judgment of experts, while different software packages may produce unstable automatic segmentation results based on the same MRI data (106, 167). Future research should focus on constructing a multimodal neuroimaging fusion framework that combines the advantages of different imaging techniques to improve the ability to identify the preclinical stage of AD, enhance disease treatment monitoring, and provide objective imaging evidence for clinical intervention.

This review systematically integrates the application and findings of neurophysiological and neuroimaging techniques in AD research. Extensive evidence indicates that the pathophysiological processes of AD exhibit characteristic alterations at multiple levels, including macrostructural, functional network, and microscopic electrical activity. These alterations provide valuable biomarkers for early diagnosis, disease progression monitoring, and mechanism exploration. The multimodal biomarkers discussed in this review closely mirror the clinical progression of the AD continuum. In the preclinical and SCD stages, functional alterations typically precede macroscopic structural damage. Driven by an underlying excitation-inhibition imbalance, this phase is characterized by Aβ-related network hypersynchronization on MEG and early hyperactivation in task-fMRI, often interpreted as an active but precarious compensatory response to initial synaptic dysfunction. Subtle changes in pre-attentive ERP components, such as the MMN, also emerge. As the disease progresses to the MCI stage, these early compensatory efforts give way to localized physiological deficits. This is evident through the attenuation of the episodic memory-related P600/LPC component, impaired cortical plasticity measured by TMS-EEG, and the onset of medial temporal lobe atrophy alongside early white matter microstructural damage. Ultimately, in the AD dementia stage, widespread network collapse occurs. This terminal phase is marked by global EEG spectral slowing, severe DMN disconnection on rs-fMRI, and the emergence of N400 deficits, indicating the spread of pathology to neocortical semantic networks. Importantly, while these neuroimaging signatures conceptually follow a sequential trajectory, clinical reality involves substantial individual heterogeneity, where pathological hypersynchronization and disconnection frequently overlap. Nevertheless, integrating these complementary modalities establishes a robust, stage-specific biomarker framework. This trajectory-based multimodal model allows for precise disease staging, capturing the full neurobiological continuum from early synaptic E-I imbalance to end-stage macrostructural and network failure.

Furthermore, advanced techniques such as MEG and fNIRS enrich the multimodal research framework. While traditional EEG excels at capturing rapid temporal dynamics, MEG, due to minimal signal distortion from the skull, provides more precise cortical source localization, enabling more accurate identification of early excitation-inhibition imbalances and regional hypersynchronization in AD. fNIRS, as a portable and cost-effective complementary technique, facilitates the assessment of prefrontal hemodynamic abnormalities and alterations in local connectivity patterns. By combining the macroscopic structural information from structural MRI, large-scale functional network mapping from fMRI, the portable cortical hemodynamic monitoring capabilities of fNIRS, and millisecond-level tracking from neuroelectrophysiological techniques, these technologies form a highly complementary multimodal framework. This integration overcomes the inherent limitations of individual modalities.

In summary, from millisecond-level synaptic activity abnormalities to second-level functional network disruptions to macrostructure brain atrophy, these multimodal neural biomarkers collectively paint a comprehensive picture of the pathological progression of AD. They not only enhance our understanding of AD as a “network disconnection syndrome” but also provide a comprehensive assessment tool that integrates functional and structural biomarkers for clinical application. With advancements in data fusion analytics and machine learning algorithms, the integration of these complementary biomarkers holds promise for earlier and more precise personalized diagnosis and treatment of AD.
